# Doxycycline-mediated effects on persistent symptoms and systemic cytokine responses post-neuroborreliosis: a randomized, prospective, cross-over study

**DOI:** 10.1186/1471-2334-12-186

**Published:** 2012-08-10

**Authors:** Johanna Sjöwall, Anna Ledel, Jan Ernerudh, Christina Ekerfelt, Pia Forsberg

**Affiliations:** 1 Clinic of Infectious Diseases, University Hospital, SE-58185, Linköping, Sweden; 2 Center for Research in General Practice, Department of Health and Society, Faculty of Health Sciences, Linköping University, SE-58185, Linköping, Sweden; 3 Division of Clinical Immunology, Department of Clinical and Experimental Medicine, Faculty of Health Sciences, Linköping University, SE-58185, Linköping, Sweden; 4 Division of Infectious Diseases, Department of Clinical and Experimental Medicine, Faculty of Health Sciences, Linköping University, SE-58185, Linköping, Sweden

**Keywords:** Neuroborreliosis, Post-treatment, Symptoms, Doxycycline, Immunomodulation, Randomized, Cytokines, Cross-over

## Abstract

**Background:**

Persistent symptoms after treatment of neuroborreliosis (NB) are well-documented, although the causative mechanisms are mainly unknown. The effect of repeated antibiotic treatment has not been studied in detail. The aim of this study was to determine whether: (1) persistent symptoms improve with doxycycline treatment; (2) doxycycline has an influence on systemic cytokine responses, and; (3) improvement of symptoms could be due to doxycycline-mediated immunomodulation.

**Methods/Design:**

15 NB patients with persistent symptoms ≥6 months post-treatment were double-blindly randomized to receive 200 mg of doxycycline or a placebo for three weeks. After a six-week wash-out period, a cross-over with a three-week course of a placebo or doxycycline was conducted. The primary outcome measures were improvement of persistent symptoms assessed by neurological examinations, a symptom severity score and estimation of the quality of life. The secondary outcome measure was changes in systemic cytokine responses.

**Results:**

All 15 patients finished the study. No doxycycline-mediated improvement of post-treatment symptoms or quality of life was observed. Nor could any doxycycline-mediated changes in systemic cytokine responses be detected. The study was completed without any serious adverse events.

**Discussion:**

No doxycycline-mediated improvement of post-treatment symptoms or quality of life was observed. Nor could any doxycycline-mediated changes in systemic cytokine responses be detected. The study was completed without any serious adverse events. To conclude, in this pilot study, doxycycline-treatment did not lead to any improvement of either the persistent symptoms or quality of life in post-NB patients. Accordingly, doxycycline does not seem to be the optimal treatment of diverse persistent symptoms post-NB. However, the results need to be confirmed in larger studies.

**Trial registration:**

NCT01205464 (clinicaltrials.gov)

## Background

Neuroborreliosis (NB), caused by the tick-borne spirochete *Borrelia* (*B*.) *burgdorferi*, is the most common form of disseminated Lyme borreliosis (LB) in Sweden [1] and Europe [2]. The clinical outcome after antibiotic treatment is fairly good [3,4]. Nevertheless, some patients have persistent symptoms post-treatment [5-9], which may include fatigue, myalgia, arthralgia, persistent facial palsy, sensory disturbances and neurocognitive dysfunctions. This phenomenon, whose pathogenesis is unknown, constitutes a challenge for the health care system, since clear diagnostic criteria are lacking [10], the symptoms do not correspond with objective measures of nervous system disease [11,12] or with laboratory measures indicating inflammation [13-15]. Hitherto, there is no evidence for a persistent *B. burgdorferi* infection [14,16], nor has resistance to recommended antibiotics been demonstrated *in vitro *[17]. A number of patients report temporary improvement of the persistent symptoms while receiving repeated treatment with doxycycline, but the symptoms tend to return a short time after end of treatment. Interestingly, in a recent review, Fallon [18] proposed that the persistent symptoms might be explained by an ongoing cytokine response, without infection, in the central nervous system (CNS). In fact, pro-inflammatory cytokines such as interleukin (IL)-6 and tumor necrosis factor (TNF), which contribute to the inflammatory process in the CNS during NB [19,20], have been experimentally shown to be involved in cognitive processes in the CNS [21]. In addition, an association between neuropsychiatric symptoms and prolonged cytokine stimulation has been demonstrated with IL-2 and interferon (IFN)-α [22]. Furthermore, previous studies by others and us have shown an inflammatory, T helper 1-type immune response both in the blood [23-25] and in the cerebrospinal fluid (CSF) [26-28] in NB patients. This immune reactivity could be altered by antibiotics, which apart from their anti-bacterial effects, are known to have anti-inflammatory properties [29]. Indeed, doxycycline has been shown to reduce the production of several pro-inflammatory cytokines, such as TNF, IL-6 and IL-8 in human monocytes, stimulated with *B. burgdorferi *[30]. Tetracyclines have also been shown to have neuroprotective properties both in *in vitro* and *in vivo* models of stroke [31,32] and in multiple sclerosis (MS) [33].

Since antibiotics have several side effects, it is important to elucidate, in a placebo-controlled manner, the relevance of a possible immunomodulating impact of doxycycline on the residual symptoms post-NB. To our knowledge, this issue has not been studied before. The aim of this hypothesis-driven, prospective study was to determine whether doxycycline has an impact on persistent symptoms post-NB, possibly through alterations in systemic immune responses.

## Methods/Design

### Participants

Eligible participants were patients with age 18–85 years with a history of NB; diagnosed according to the European guidelines [34], with persistent symptoms ≥6 months post-treatment, such as fatigue, facial palsy, headache, radiculitis and cognitive and neurological dysfunctions. The exclusion criteria consisted of systemic immunosuppression (corticosteroids or other immunosuppressive drugs), a current infection or ongoing antibiotic treatment, allergy to doxycycline, pregnancy, lactation, psychiatric disorders, multiple sclerosis, rheumatoid arthritis, diabetes mellitus type I or II, systemic inflammatory diseases, liver or kidney dysfunction, current malignancy and treatment with Didanosine, Quinapril or antacids.

### Study design and settings

We conducted a prospective, randomized, double-blind clinical trial with a cross-over design. The trial was conducted at the Clinic of Infectious Diseases, University Hospital, Linköping, Sweden, and the immunological assays were performed at the Unit for Autoimmunity and Immune Regulation (AIR), Division of Clinical Immunology, the Faculty of Health Sciences, Linköping University, Sweden. The trial started in February 2005 and was completed in February 2008.

### Interventions

**Figure 1:**
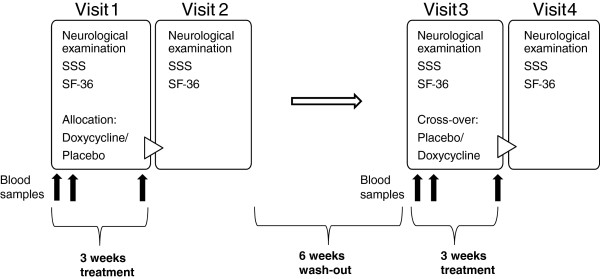
** Overview of the study interventions.** The four boxes illustrate the visits to the doctor, and the content in the boxes include the different outcome measures. At visit 1, the patients were randomly and double-blindly assigned to receive either doxycycline or a placebo treatment during 3 weeks. At the third visit, the patients received the opposite study drug, in a cross-over manner. The vertical black arrows indicate time points of blood sampling for analysis of cytokine responses in blood (at the start of, after 5 days, and at the end of treatment, respectively). During the six-week wash-out period, no assessments or examinations were carried out. Abbreviations: SSS, symptom severity score; SF-36, Short Form-36.

At the first visit, the participants underwent a neurological examination according to the study protocol. Prior to treatment, the persistent symptoms were defined and their severity score assessed according to a 10-graded symptom severity score (SSS), and the quality of life (QOL) was estimated using a Swedish version of the standardized and evaluated short-form health survey (SF-36) [35]. Furthermore, blood samples were drawn for analysis of cytokine responses. The patients were randomized in a double-blind, cross-over fashion to receive either doxycycline (2 capsules á 100 mg doxycycline monohydrate) or a placebo (2 capsules á 100 mg of cellulosum microcristallinum), once daily, for three weeks. The placebo was matched to the study drug with respect to taste, color and appearance. The persistent symptoms were assessed, using the SSS, every day during treatment. After 5 days of treatment, blood samples were drawn for analysis of cytokine responses. At the end of each treatment set, new blood samples were drawn, neurological examinations were carried out and the QOL was estimated (Figure 1). The two treatment sets were separated by a six-week wash-out period, during which time no assessments or examinations were carried out.

### Neurological examinations

The neurological examinations were carried out according to a protocol, by one of the two investigators (JS or PF), before start of and at the end of doxycycline and placebo treatment, respectively. The examination protocol contained the following parts: evaluation of neck stiffness, the finger-nose test, Romberg’s test, Grasset’s sign, walking on the heels and toes, assessment of eye movements, evaluation of nystagmus and cranial nerve function (especially the facial nerve), sensory examination, deep tendon reflexes, the Babinski plantar response, muscle strength in the extremities, and fundoscopy. The outcome of the different examinations was numerically defined as deviant (=2), if one or more of the neurological examination parts were abnormal, otherwise as normal (=1). The difference (+, -, 0) between the outcome of the examination after and before treatment with doxycycline and placebo was calculated and used for comparison.

### Assessment of symptom severity

At the first visit to the doctor, the patient’s symptoms were defined and assessed by the patient and the numerical severity level written in a form according to a 10-graded symptom severity score (SSS): 0 = no symptoms, 1–2 = mild, 3–4 = mild-moderate, 5–6 = moderate, 7–8 = severe, and 9–10 = unbearable symptoms. The symptoms were assessed at the same time every day during the three-week course of treatment with doxycycline and placebo, respectively. The difference between the SSS after and before the start of treatment was calculated for the symptoms with the two highest SSS (symptom 1 and 2, Table 1) and used for statistical comparison.

**Table 1:** Characteristics of the included patients

**No**	**Sex**	**Age (years)**	**Symptom duration before initial treatment (weeks)**	**CSF total white cell count x 10 6/L**	**Initial treatment of NB**	**Re-LP [months after initial LP] (CSF white cell count x 10 6/L)**	**Randomization**	**Symptom duration at study inclusion (months)**	**Symptoms at study inclusion**
1	F	48	4	300	Doxycycline, Ceftriaxone	1 (33)	Placebo, Doxycycline	82	Headache, myalgia, arthralgia, concentration and memory difficulties, depression, insomnia**
2	M	60	7	170	Doxycycline	2 (30)	Doxycycline, Placebo	25	**Fatigue** (1), **muscle weakness** (2), headache, arthralgia, insomnia
3	M	38	8	20	Doxycycline	12 (0.5)	Doxycycline, Placebo	57	Paresthesia, sound sensitivity, imbalance, depression**
4	M	70	2	560	Doxycycline, Ceftriaxone	10 (2.0)	Doxycycline, Placebo	87	**Hearing loss** (1), **discomfort of facial palsy** (2), arthralgia, myalgia
5	F	59	7	570	Doxycycline, Ceftriaxone	0.75 (100)	Placebo, Doxycycline	51	**Neck pain** (1), **imbalance** (2), paresthesia, headache
6	F	54	3	850	Ceftriaxone	1 (20)	Doxycycline, Placebo	27	**Hyperesthesia** (1), **eye twitching** (2)
7	F	60	3	500	Doxycycline, Ceftriaxone	2 (8.0)	Placebo, Doxycycline	75	**Hearing loss** (1), **imbalance** (2), headache, hypoesthesia
8	M	44	3	N.A♯	Doxycycline, Ceftriaxone	8 (3.0)	Placebo, Doxycycline	102	**Arthralgia** (1), **insomnia** (2), headache, discomfort of facial palsy, paresthesia, muscle weakness, concentration and memory difficulties, depression
9	M	64	12	0.7♯♯	Doxycycline	N.A	Placebo, Doxycycline	11	**Imbalance** (1)
10	F	73	5	200	Doxycycline	N.A	Doxycycline, Placebo	13	**Headache** (1), **fatigue** (2)
11	M	65	13	190	Doxycycline, Ceftriaxone	0.5 (30)	Doxycycline, Placebo	11	**Paresthesia** (1), **neck pain** (2), headache, myalgia, concentration difficulties, irritability
12	M	82	3	130	Doxycycline	N.A	Placebo, Doxycycline	11	**Imbalance** (1), **back pain** (2), headache, hearing loss, myalgia
13	M	48	4	200	Doxycycline	5 (3.0)	Doxycycline, Placebo	24	**Dysestesia** (1), **fatigue** (2), headache, hearing loss, myalgia, concentration and memory difficulties
14	M	50	3	220	Doxycycline	N.A	Doxycycline, Placebo	16	Paresthesia (1), pain in extremity/radiculitis (2)
15	F	71	3	11*	Doxycycline	N.A	Placebo, Doxycycline	13	**Discomfort of bilateral facial palsy** (1), **paresthesia** (2)

The persistent symptoms include all the declared symptoms at inclusion, of which the ones in bold with numbers in parenthesis indicate the two symptoms with the highest symptom severity scores. F, female; M, male; No, number; CSF, cerebrospinal fluid; NB, neuroborreliosis; LP, lumbar puncture; N.A, not analyzed; ♯Only the intrathecal anti-borrelia antibody index was analyzed. ♯♯ LP was done one week after initiation of treatment. * LP was done 15 weeks after initiation of antibiotic treatment. ** Symptom severity score was not assessed.

### Estimation of the quality of life

The estimated QOL was assessed according to the standardized Swedish version of the SF-36 health survey (IQOLA SF-36 Standard Swedish Version 1.0) [35] before the start of and after doxycycline and placebo treatment. The SF-36 includes eight subscales that measure physical functioning, physical limitations on usual role-related activities, bodily pain, general health perceptions, vitality, social functioning, emotional limitations on usual role-related activities, and mental health. The subscales constitute the basis for calculation of the summary scores of the mental (MCS) and physical (PCS) components of the SF-36 [36] (IQOLA SF-36 v.1, the HRQL-group, Section of healthcare research, University of Gothenburg and Sahlgrenska University Hospital, Gothenburg, Sweden). The scores range from 0 (worst) to 100 (best). The difference between the summary scores after and before the start of doxycycline and placebo treatment was used for statistical comparison.

### Preparation of peripheral blood mononuclear cells

Peripheral blood mononuclear cells (PBMC) were separated from heparinized peripheral blood using density gradient centrifugation on Lymphoprep (Medinor AB, Stockholm, Sweden), as previously described by Boyum [37]. The cells were re-suspended in tissue culture medium (TCM), as described by Forsberg *et al.*. [38], to a final concentration of 1 x 10^6^ PBMC/ml.

### Cytokine responses detected by the ELISPOT assay

The enzyme-linked immunospot (ELISPOT) assay, as previously described [39], was used for analysis of unstimulated (spontaneous) and antigen-stimulated cytokine secretion in PBMC, using the following antigens: an outer surface protein–enriched fraction (OF) of *B. garinii* strain Ip90 (10 μg/ml), as previously described [40] and an influenza-vaccine ([INFL], 2002/2003, dilution 1:1000, batch: W6287-2, SBL Vaccine Distribution, Stockholm, Sweden), used as reference antigens for recall responses and phytohemagglutinin ([PHA] 20 μg/ml, Sigma Aldrich, MO, USA), used as a positive control. In short, the ELISPOT plates were coated with monoclonal antibodies, mouse anti-human IL-4, IL-12p70, IFN-γ (Mabtech AB, Stockholm, Sweden) and transforming growth factor (TGF)-β (R&D Systems, MN, USA), diluted with sterile phosphate buffered saline ([PBS], Medicago AB, Uppsala, Sweden) to a final concentration of 15 μg/ml. The plates were incubated overnight at 4°C and frozen at −20°C for a maximum of 3 months. At analysis, the plates were thawed at room temperature (RT), unspecific binding sites were blocked with TCM followed by addition of 100 000 PBMC/well. Thereafter, either TCM (spontaneous secretion) or OF was added to the wells in triplicate, and INFL or PHA in duplicate. TCM was used as a negative control. The cells were cultured undisturbed for 48 h at 37°C, 5% CO_2_ with 95% humidity. Developing of spots, representing cytokine secreting cells, was conducted with matched biotin-conjugated monoclonal antibodies, mouse anti-human IL-4, IL-12p70, IFN-γ (Mabtech AB) and TGF-β (R&D Systems), diluted in PBS-Tween (Medicago AB, Uppsala, Sweden) to 1 μg/ml. After incubation in a dark humidity chamber at RT, alkaline phosphatase-conjugated streptavidin, diluted 1:1000 in PBS-Tween, was added. The final step included addition of nitro blue tetrazolium and 5-bromo-4-chloro-3-indolyl phosphate, diluted in alkaline phosphatase (AP)-buffer (AP conjugate substrate kit, Bio-Rad, Hercules, CA, USA). The spots were counted with the AID EliSpot Reader System (software version 3.2.2, Germany). One spot was equivalent to one cytokine-secreting cell per 100 000 lymphocytes. The median of the triplicates was used for analysis of the number of cytokine secreting cells. To determine the OF-specific secretion, the number of spots in the unstimulated wells was subtracted from the number in the OF-stimulated wells. The OF-specific secretion of IFN-γ and IL-4 was positive for values >15 spots/100 000 cells and >10 spots/100 000 cells, respectively, according to previous results [41].

### Antigen-stimulation of peripheral blood mononuclear cells

PBMC, suspended in TCM to a concentration of 1 x 10^6^ cells/ml, were incubated for 48 h at 37°C, 5% CO_2_ with 95% humidity, with 1 ml of either lipopolysaccharide ([LPS], from *E. coli* 026:B6, Lot nr: 123 K4021, 0.2 μg/ml, Sigma Aldrich, MO, USA), PHA (20 μg/ml, Sigma Aldrich) or OF (10 μg/ml); all diluted with TCM. PBMC incubated with merely TCM were used for analysis of spontaneous cytokine secretion. After incubation, the cells were centrifuged at RT, 500× *g* for 10 min. The cell supernatants were stored at −70°C.

### Analysis of cytokine levels in serum and in cell supernatants

Levels of IL-1β, IL-2, IL-4, IL-5, IL-6, IL-8, IL-10, granulocyte macrophage colony stimulating factor (GM-CSF), IFN-γ and TNF were analyzed in serum using a Human Ultrasensitive Cytokine 10-plex kit (LHC6004, Invitrogen, CA, USA), according to the manufacturer’s instructions. All cytokines were analyzed undiluted. Levels of IL-6 and IL-8 in PBMC supernatants were analyzed using single-bead kits (LHC0061 and LHC0081, Invitrogen), whereas a six-plex kit was used (with an extracellular protein buffer reagent kit [LHB0001], Invitrogen) for detection of IL-1β (LHC0011), IL-4 (LHC0041), IL-10 (LHC0101), IFN-γ (LHC4031), GM-CSF (LHC2011) and TNF (LHC3011, all from Invitrogen) in the cell supernatants. Both IL-6 and IL-8 were diluted 1:100, whereas the other cytokines were analyzed undiluted. StarStation v.3.0 (Applied Cytometry, Sheffield, UK) was used for data acquisition and analysis. Values below the lowest value of the standard curve were assigned half the value of the lowest standard point.

### Outcomes

The primary endpoints were a statistically significant doxycycline-mediated improvement of the persistent symptoms, with respect to; a) the neurological examinations, b) the assessment of symptom severity, and/or; c) the estimated QOL. The secondary endpoint was a statistically significant doxycycline-mediated effect on systemic cytokine responses.

### Sample size

**Figure 2:**
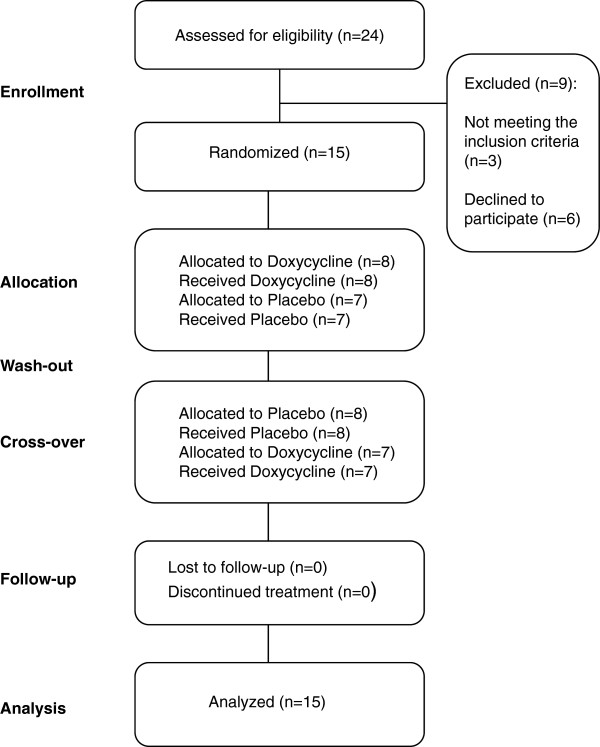
** The study flow chart.** Abbreviations: n, number.

Twenty-four patients, who were previously examined and treated for well-diagnosed NB at the Clinic for Infectious Diseases in Linköping and of whom several had a continued follow-up at the clinic, were initially identified during autumn 2004, through screening of the entire catchment-area in the County of Östergötland, and were asked by phone to participate in the study. Nine of them were excluded prior to the randomization, whereof three were without current symptoms and six declined to participate due to reasons that went together with the study arrangement (Figure 2).

### Randomization

Independent pharmacists at the Pharmacy’s Production and Laboratories (Stockholm, Sweden) created a computer-generated, anonymous randomization schedule for all participants, and they manufactured the study drugs. The drug bottles were consecutively numbered for each patient, according to the schedule. Each patient was assigned a number by one of the two clinical investigators (JS or PF), and received the drug by the responsible research nurse, who was delegated the responsibility for storage and administration of the study drugs. The randomization code, which was stored in a locked closet at the Pharmacy of Linköping University Hospital, was kept undisclosed in a sealed envelope throughout the study. The code was broken only when the study was finished and all raw data had been compiled and saved in locked data files.

### Ethics

The study was approved by the Regional Ethical Review Board at Linköping University, Sweden. Written informed consent was obtained from all the participants. Regular inspections were performed by an independent monitoring group (the TEKLA-group) at the University Hospital, Linköping. Good clinical and laboratory practice were applied throughout the study.

### Statistical methods

Since data were in general not normally distributed, non-parametric tests were used for the statistical calculations. The Wilcoxon rank sum test was used for calculation of intra-individual differences in change of neurological examinations with doxycycline and placebo treatment, respectively, whereas the Mann Whitney *U* test was used for a corresponding comparison between the treatment groups. The Wilcoxon rank sum test was also used for analysis of intra-individual differences in change of the two symptoms with the highest SSS, the PCS and MCS scores and of cytokine responses during doxycycline and placebo treatment, whereas Mann Whitney *U* test was used for corresponding comparisons between the two groups. As a precaution, a parametric test (ANOVA) was also used, without finding any significant differences in cytokine responses between the two groups. Statistical calculations were made with the PASW Statistics 18 software for Microsoft (SPSS Inc., Chicago, IL, USA). Graphs were made with GraphPad Prism, version 5.02 for Microsoft (GraphPad Software Inc., San Diego, CA, USA). A p-value ≤0.05 was considered significant.

## Results

### Clinical characteristics

15 patients (6 women, 9 men) with a history of NB were included in the study. The mean age was 59 years (range 38–82). The median pre-treatment symptom duration was 4 weeks (range 2–13) and the median CSF cell count at the time of diagnosis of NB was 200 x 10^6^ leucocytes/L (range 0.7–850), of which a majority were mononuclear cells. Various persistent symptoms were reported, although most of the patients had headache, fatigue, sensory disturbances, arthralgia and myalgia. All 15 patients fulfilled the criteria for post-Lyme disease syndrome, as defined by the Infectious Diseases Society of America [42]. The mean duration of the post-treatment symptoms was 3.4 years (range 0.9–8.5) at inclusion. All the patients had received treatment of NB with either doxycycline or intravenous ceftriaxone for 10–14 days, according to the Swedish treatment recommendations [43]. A re-lumbar puncture, prior to inclusion, had been done as part of clinical routine in ten of the patients, with signs of diminished or normal cell counts in the CSF analysis. However, most of the patients had been treated with at least one additional course of either oral (doxycycline) or intravenous (ceftriaxone) antibiotics, due to persistence of the symptoms. At the first treatment set, eight patients were randomized to receive doxycycline and seven to receive a placebo, with a cross-over during the second treatment set (Table 1).

### Neurological examinations

In cases of a deviant neurological examination at baseline or at the follow-ups, the facial nerve, the balance, the sensibility or tendon reflexes were affected. None of the patients developed new, objective neurological deficits or experienced worsening of current neurological signs during the study period. Four patients improved with regard to Romberg’s test, whereof one improved during doxycycline-treatment and three during placebo-treatment (Table 2). Most of the patients had unchanged outcome on neurological examination throughout the study (Table 2). No significant changes in neurological examinations were found either within or between the doxycycline and placebo group.

**Table 2:** Outcome of the neurological examinations, the symptom severity score and assessment of quality of life with doxycycline and placebo treatment

	**Doxycycline 3 weeks**			**Placebo 3 weeks**		
**Patient no.**	**Neurological examination**	**SSS symptom 1/2**	**SF-36 PCS/MCS**	**Neurological examination**	**SSS symptom 1/2**	**SF-36 PCS/MCS**
1	0	N.A	+/+	0	N.A	−/−
2	+	+/+	N.A	0	-/0	+/+
3	0	N.A	−/+	0	N.A	N.A
4	0	0/0	+/−	0	0/0	N.A
5	0	+/−	+/−	0	0/+	0/+
6	0	0/+	−/−	0	0/0	N.A
7	0	0/+	N.A	0	0/-	−/+
8	0	0/+	+/−	0	+/+	−/+
9	0	+/N.A	+/+	0	-/N.A	−/+
10	0	+/+	+/+	+	0/+	−/+
11	0	0/0	−/+	+	0/0	−/+
12	0	0/0	−/−	0	0/+	+/−
13	0	-/0	−/+	+	-/0	+/−
14	0	0/N.A	−/+	0	0/N.A	N.A
15	0	0/0	+/−	0	0/0	+/+

A difference between the results of the examinations and assessments after and before treatment was used for comparison of the outcome of treatment, and was defined as: +, improvement; 0, unchanged or –, worse. For the SSS, the two symptoms (symptom 1 and 2) with the highest severity scores were chosen for comparison. No significant differences were found in any of the outcome measures either within or between the doxycycline and placebo treatment groups. no, number; SSS, symptom severity score; SF-36, Short Form-36; PCS, physical component summary; MCS, mental component summary; N.A, not analyzed.

### Assessment of symptom severity

With doxycycline treatment, four (no. 2, 5, 9, 10) patients improved with respect to the symptom with the highest severity score (spt1), whereas one (no. 8) patient improved with respect to spt1 with placebo-treatment, without any significant differences between the groups (Table 2). Five (no. 2, 6, 7, 8, 10) patients improved with doxycycline treatment with respect to the other one of the two symptoms (spt2) with the highest severity scores, whereas four (no. 5, 8, 10, 12) improved with placebo-treatment, without any significant differences between the groups. In all, two patients got worse regarding one of the symptoms with doxycycline treatment, and four with placebo treatment (Table 2). However, no significant differences were found when comparing changes in severity of the two symptoms within or between the doxycycline and placebo group.

### Estimation of the quality of life

The PCS improved in seven (no. 1, 4, 5, 8, 9, 10, 15) patients with doxycycline treatment and in four (no. 2, 12, 13, 15) with placebo-treatment (Table 2). The MCS improved in seven (no. 1, 3, 9, 10, 11, 13, 14) with doxycycline treatment and in eight (no. 2, 5, 7, 8, 9, 10, 11, 15) with placebo-treatment. Six patients (no. 3, 6, 11, 12, 13, 14) got worse in PCS with doxycycline treatment, and six (no. 1, 7, 8, 9, 10, 11) with placebo. The MCS got worse in six patients (no. 4, 5, 6, 8, 12, 15) with doxycycline treatment, and in three (no. 1, 12, 13) with placebo (Table 2). No significant differences were found in change of PCS or MCS either within or between the two treatment groups. Notably, only one patient had unchanged QOL, during placebo treatment (Table 2). However, in comparison with the general Swedish population (matched for mean age) [44], the patients had significantly decreased mean PCS at time of inclusion (*p* = 0.002).

### Cytokine responses detected by the ELISPOT assay

**Figure 3:**
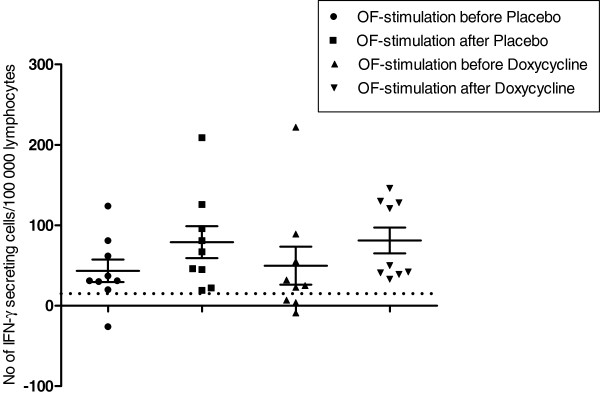
**The number of OF-specific IFN-γ secreting mononuclear cells/100 000 lymphocytes, detected with the ELISPOT assay, before and after treatment with Doxycycline and Placebo.** The graph illustrates cytokine data from the first nine included patients. The OF-specific cytokine secretion was obtained by subtracting the number of spots in unstimulated (spontaneous) wells from the number of spots in OF-stimulated wells, illustrating the Borrelia-specific memory immune response. Values, which represent medians of triplicates, are given as number of cytokine secreting cells/100 000 lymphocytes. The dotted horizontal line indicates the cut-off (= 15 cells/100 000) for OF-specific IFN-γ secretion. No significant differences in change of the number of cytokine secreting cells were found either within or between the two treatment groups. Horizontal lines denote the median. Abbreviations: OF, outer surface protein–enriched fraction of B. garinii strain Ip90; IFN, interferon; ELISPOT; enzyme-linked immunospot assay.

The analysis included data from the first nine patients (patient number 1–9, Table 1), since the assays of the last six patients were omitted due to technical problems related to switch of batch for the micro titer plates. Most patients had an OF-specific IFN-γ response (Figure 3), whereas few patients had an OF-specific IL-4 response (data not shown). Likewise, the number of IL-12p70 and TGF-β secreting cells was low (data not shown). No significant differences were found in either spontaneous or antigen (OF and INFL)-stimulated IFN-γ, IL-4, IL-12p70 or TGF- β responses within or between the doxycycline and placebo treatment groups.

### Cytokine levels in serum

The median values of the circulating levels of IL-1β, IL-6, IL-8, IL-10 and GM-CSF in serum before and after doxycycline and placebo treatment are presented in Table 3. The median of the levels of IL-2, IL-4, IL-5, IFN-γ and TNF were low or undetectable (data not shown). No significant differences in change of the circulating cytokine levels in serum were found either within or between the doxycycline and placebo treatment groups (data not shown).

**Table 3:** Cytokine levels in serum and in cell supernatants before and after doxycycline and placebo treatment

**Cytokine**	**Antigen**	**Doxycycline median pg/ml (Interquartile range)**		**Placebo median pg/ml (Interquartile range)**		**Detectable (%)**
		**Before**	**After**	**Before**	**After**	
**Serum**						
IL-1β		0.43 (0.13-1.50)	0.60 (0.13-1.10)	0.52 (0.13-0.70)	0.60 (0.13-0.84)	68
IL-6		0.19 (0.19-1.60)	0.19 (0.19-2.70)	0.19 (0.19-2.70)	0.19 (0.19-3.20)	43
IL-8		8.15 (6.0-12.9)	9.78 (7.70-14.3)	10.6 (7.40-14.9)	10.1 (7.90-13.7)	100
GM-CSF		0.75 (0.75-9.85)	0.75 (0.75-9.10)	0.75 (0.75-10.2)	0.75 (0.75-11.5)	40
IL-10		0.15 (0.15-1.80)	0.15 (0.15-1.72)	0.15 (0.15-1.30)	0.15 (0.15-1.97)	45
**Supernatants**						
IL-1β	OF	267 (147–416)	276 (178–353)	258 (160–372)	257 (212–325)	100
	LPS	373 (297–578)	336 (273–459)	384 (306–464)	376 (272–417)	100
IL-6	OF	8495 (5577–11552)	10018 (7283–11436)	9017 (7893–12936)	10549 (6765–11870)	97
	LPS	7457 (5522–9344)	7043 (6173–9401)	7339 (5640–9010)	7129 (6164–8376)	100
IL-8	OF	234726 (182996–353519)	256238 (224077–323425)	264450 (187637–377221)	254926 (343734)	100
	LPS	170005 (84896–191690)	158091 (116470–210940)	159121 (119796–175857)	153546 (203419)	100
	unstim.	949 (425–3337)	887 (412–2657)	966 (580–2637)	728 (443–1324)	100
TNF	OF	49.4 (41.8-72.4)	51.8 (26.9-210)	91.2 (66.7-141)	69.3 (25.8-152)	77
	LPS	45.0 (23.8-61.3)	45.4 (31.7-56.4)	36.7 (27.1-57.5)	37.1 (26.4-51.9)	77
IL-10	OF	50.6 (41.8-84.7)	59.3 (48.4-84.7)	64.4 (46.3-100)	67.3 (47.0-86.3)	82
	LPS	177 (130–272)	184 (114–259)	201 (154–255)	181 (119–280)	98

The table shows median values for spontaneous cytokine levels in serum and antigen-stimulated (and unstimulated IL-8 levels) cytokine levels in peripheral blood mononuclear cell supernatants, before and after doxycycline and placebo treatment from all 15 included patients. IL, interleukin; GM-CSF, granulocyte macrophage colony-stimulating factor; TNF, tumor necrosis factor; OF, outer surface–enriched fraction of *B. garinii* strain Ip90; LPS, lipopolysaccharide; unstim., unstimulated.

### Cytokine levels in mononuclear cell supernatants

In general, the concentrations of IL-4, IFN-γ and GM-CSF were low and detectable only in PHA-stimulated samples, whereas IL-1β, IL-6, IL-8, IL-10 and TNF were detectable in most of the OF and LPS-stimulated samples and, except for IL-8, undetectable in unstimulated samples. Both LPS- and OF-stimulated levels of IL-1β, IL-6, IL-8, IL-10 and TNF were high both before and after treatment with doxycycline and a placebo (Table 3). However, no significant differences were found in change of either antigen-stimulated or spontaneous cytokine levels within or between the doxycycline and placebo treatment groups.

### Safety

No serious adverse events occurred during the study time. However, during doxycycline treatment, nausea was reported by four patients and vomiting and abdominal pain by one patient each. One doxycycline-treated patient (no. 10) suffered from a moderate facial skin erythema at the end of the first treatment set, but she continued the study without any interruption in the treatment. During placebo treatment, only one patient reported an adverse event (abdominal pain).

## Discussion

This randomized, hypothesis-driven pilot study could not demonstrate any significant doxycycline-mediated improvement of the neurological status, severity of the persistent symptoms or the QOL in NB patients with persistent symptoms post-treatment. Nor was any specific impact of doxycycline on cytokines secreted by PBMC or on cytokine levels in serum found. Any significant placebo effect could neither be demonstrated. Several studies have highlighted the occurrence of subjective complaints after treatment of LB [45-47]. However, the case definitions often differ, which complicates comparison of the results. Many of the reported symptoms are also common in the general population [48], and have *de facto* been shown to occur as frequently in controls without LB [49-51]. In this trial, the study population consisted of well-characterized patients with a history of NB, and they had received treatment with doxycycline or ceftriaxone at the time of diagnosis. Treatment failure is unlikely an explanation to the persistent symptoms, since both treatment alternatives have been shown to be equally effective with respect to clinical outcome [52-55]. Moreover, doxycycline has been shown to penetrate well into the CSF and concentrations above the estimated minimum inhibitory concentration for *B. burgdorferi* are exceeded with a daily dose of 200 mg [56,57]. Interestingly, in a recent Norwegian study on NB, a pre-treatment symptom duration ≥ 6 weeks, a high pre-treatment CSF cell count and female gender were considered as risk factors for development of remaining complaints one year post-treatment of NB [58]. In our study, five patients had a pre-treatment symptom duration exceeding 6 weeks and the median total CSF cell count was even higher than in the Norwegian study, indicating a strong inflammatory response in the CSF.

Most of the patients had an unchanged neurological status throughout the study, with no significant differences between the two treatments. Several of the reported symptoms were of neurocognitive character and it is possible that even subtle changes in these symptoms would better have been detected, if validated neurocognitive or psychiatric tests would have been used in addition to the SSS. The SSS was designed by the authors in order to observe changes in symptom severity during treatment. However, it is not a validated test, which makes comparison with other reports difficult. A majority of the patients had unchanged symptom severity, without any significant differences between the treatment sets.

All included patients had a post-treatment symptom duration by far exceeding 6 months, with a large range in duration. The phenomenon of persistent symptoms post-treatment of LB has in recent years been named Post-Lyme disease syndrome [15,53,59,60], and a definition of the inclusion and exclusion criteria has been proposed by the Infectious Diseases Society of America [42]. All the 15 patients included in this study fulfilled these criteria. Most of the patients had been retreated with another course of antibiotics, without long-standing improvement of the symptoms. At inclusion, both the MCS and PCS were decreased in the study population and the PCS was significantly decreased compared with the general Swedish population, indicating a reduced QOL in the study population. Similar results were obtained in a Norwegian follow-up study of patients previously treated for NB, in which comparison was made with matched healthy controls [61]. However, in our study neither the PCS nor the MCS changed significantly during three weeks of doxycycline or placebo treatment. It is not likely that a longer treatment duration would have revealed any differences in QOL, since similar results were obtained in a study of *B. burgdorferi*-seropositive patients with persistent musculoskeletal and neurocognitive symptoms >6 months after treatment, in which no significant differences in QOL were observed between patients receiving intravenous Ceftriaxone for 30 days, followed by oral doxycycline for 60 days and those receiving a placebo [14].

The spontaneous, circulating cytokine levels in serum were in general low, except for IL-8, which could be detected in all samples, but without any significant changes during doxycycline and placebo treatment. A treatment duration of three weeks was, in accordance with previous studies [32,62], assumed to be sufficient to influence systemic cytokine responses, whereby any doxycycline-mediated changes in cytokine levels could be detected with the sensitive assays. A significant reduction in vascular IL-6 expression and reduced protein levels of IL-8, IL-13 and GM-CSF have been observed already after two weeks of doxycycline treatment (50–300 mg/day) in patients scheduled for aneurysm surgery [63]. IL-1β, IL-6, IL-8, IL-10 and TNF were all well detectable in antigen-stimulated PBMC-supernatants, but no significant doxycycline-mediated changes could be observed. The majority of the patients had a substantial OF-specific IFN-γ response, which is in line with other studies [26,38], whereas the number of OF-specific IL-12p70-, TGF-β- and IL-4-secreting cells was low. From this study we cannot exclude any doxycycline-mediated changes in cytokine responses in the CSF. Since cytokine responses in NB mainly are compartmentalized to the CSF [39], it would have been relevant to measure cytokine levels in the CSF *per se*. However, this was not feasible from an ethical point of view.

The strengths of this study are the well-characterized patients without drop-outs, the randomized, placebo-controlled, cross-over design with a long wash-out period between the treatment sets, and several clinical and immunological evaluations. The limitations include restrictive exclusion criteria and a time-consuming study protocol, which made the enrolment of patients difficult, and consequently the patient number small. For this reason, the results need to be assessed with caution. Further, no blood analyses of drug concentrations were carried out, to assure that the study drugs were taken according to prescription. However, all the patients were anxious to participate in the study, they assured that they followed the prescriptions, and they were aware that they could disrupt the treatment anytime in consultation with the investigators. More patients reported adverse events during doxycycline treatment, than during treatment with a placebo. Despite this fact, no significant differences in the subjective assessments were found between the two treatments.

The patient, who suffered from a facial erythema at the end of the doxycycline treatment, was allowed to continue the study, since the symptoms were judged as mild to moderate, and they declined rapidly. However, this side effect might have influenced the further assessments by this patient. Retrospectively, it may be noted that the subjective assessment of the symptom severity and QOL were not significantly affected by that event.

## Conclusions

In this pilot study, we could not demonstrate any doxycycline-mediated improvement of neither post-treatment symptom severity nor QOL in patients with persistent symptoms after adequate antibiotic treatment of NB or any evidence of immunomodulating effects of doxycycline on systemic cytokine responses in these patients. Consequently, doxycycline does not seem to be the optimal treatment of persistent symptoms post-NB. However, the results need to be confirmed in larger studies. In addition, studies addressing alternative treatment approaches for post-treatment symptoms are requested.

## Competing interests

The authors declare that they have no competing interests.

## Authors’ contributions

JS, PF, CE and JE designed the study. JS and PF enrolled, evaluated and treated the participants. JS, AL and PF carried out the data collections and statistical analyses. JS and PF drafted the paper. All authors read and approved the final manuscript.

## Pre-publication history

The pre-publication history for this paper can be accessed here:

http://www.biomedcentral.com/1471-2334/12/186/prepub
